# Trajectory priming through obstacle avoidance in motor imagery – does motor imagery comprise the spatial characteristics of movement?

**DOI:** 10.1007/s00221-024-06951-3

**Published:** 2024-12-02

**Authors:** James W. Roberts, Caroline J. Wakefield, Robin Owen

**Affiliations:** 1 Brain & Behaviour Research Group, Research Institute of Sport & Exercise Sciences (RISES), Liverpool John Moores University, Tom Reilly Building, Byrom Street, Liverpool, L3 5AF UK; 2 School of Health and Sport Sciences, Liverpool Hope University, Hope Park, Liverpool, L16 9JD UK

**Keywords:** Functional equivalence, Sensory feedback, Forward model, Mental chronometry, Aiming

## Abstract

Motor imagery and execution often indicate a similar trend in the temporal characteristics of movements. This finding supports the notion of functional equivalence, whereby imagery and execution use a common neural representation. However, there is comparatively limited evidence related to the spatial characteristics of movements; no doubt owing to the absence of an actual spatial trajectory during imagery. Therefore, we adapted the trajectory priming paradigm involving an obstacle, where the trajectory adopted in a trial (*n*) is directly contaminated by a previous trial (*n*-1). If imagery accurately represents the spatial characteristics, then we would predict a similar priming effect as execution. Participants completed a series of trial blocks under different imagery/execution protocols, where the test trial (*n*) comprised execution alone, while the previous trial (*n*-1) involved imagery or execution. Each block comprised pairs of trials with alternate or consistent presentations of a virtual obstacle (O) or no obstacle (N): N-N, N-O, O-N, O-O. For trial *n*-1 (imagery/execution), there was a more prolonged reaction and movement time for imagery compared execution. Most importantly for trial *n* (execution), there was an increase in early angular and peak deviation following an obstacle compared to no obstacle in trial *n*-1, but only when it was execution and not imagery. These findings suggest imagery holds a limited representation of the spatial characteristics, while functional equivalence may be limited to the temporal characteristics.

**Supplementary Information:**

The online version contains supplementary material available at 10.1007/s00221-024-06951-3.

## Introduction

Motor imagery refers to the mental rehearsal of movement in the form of a visual (“see”) and/or kinaesthetic (“feel”) simulation without any overt physical execution (Vealey and Walter 1993; for a glossary of imagery terms and categories, see Moreno-Verdú et al. 2024). It is commonly used to enhance motor performance (Ladda et al. 2021) and (re-)learn motor skills (Malouin et al. 2010). The value and vividness attributed to motor imagery has been primarily explained by the functional equivalence theory (Jeannerod 1994, 1999), where the neural representations that are responsible for the imagery and execution of movement are deemed to be the same or similar. This theory has been heavily supported by neuro-imaging (Schubotz and von Cramon 2004; Filimon et al. 2007; Hétu et al. 2013) and brain stimulation (Fadiga et al. 1999; Wright et al. 2018) studies, whereby the neural structures that are responsible for execution are known to be similarly activated or used within imagery.

From a behavioural perspective, the main source of evidence that has been leveraged in support of this theory has been the mental chronometry paradigm, whereby individuals are tasked with imagining a particular movement (e.g., discrete manual aiming movement) at varying levels of difficulty (i.e., low difficulty with large size and/or short amplitude target, high difficulty with small size and/or long amplitude target) (Decety et al. 1995; Sirigu et al. 1996; Gueugneau et al. 2008, 2017; Rozand et al. 2015) (for a review, see Guillot et al. 2012). In this regard, the time of imagined responses is typically indexed by a series of micro-movements that each represent the start and end of a movement, while still remaining physically in a set location (e.g., press/release of a button over the start position). Findings indicate that the positive relation between difficulty and time for executed movements also extends to imagined movements─thus abiding by *Fitts’ Law* (Fitts 1954; Fitts and Peterson 1964) (i.e., more difficult task taking a longer time to complete, and vice versa).

While highly informative, it is clear to see that the fore mentioned evidence is somewhat limited to the temporal domain with next to no evidence surrounding the spatial characteristics, including the movement trajectory (e.g., displacement and direction of the moving limb). This research lacuna is largely a result of imagery often not involving an overt physical movement, where a shift in position could be otherwise reasonably detected. Alternatively, there are some researchers that have attempted to make a post-trial retrospective account of estimated spatial errors as a proxy to the spatial characteristics potentially within imagery. Indeed, it has been shown that the estimated imagined and actually executed spatial errors positively correlate with one another for a dart-throwing (Dahm and Rieger 2019) and novel manual tracing task (Ingram et al. 2022). These findings have been explained by the generation of an internal forward model, which can effectively substitute or emulate the sensory feedback that is otherwise absent during imagery (Grush et al. 2004; Rieger et al. 2023). Specifically, an efference copy can be adapted without overt motor outflow for motor imagery to form a prediction regarding the sensory consequences, which when compared with the desired state (i.e., intended outcome), provides at least some indication of where the limb should be in space.

However, there is also evidence of a discrepancy in the bias of estimated imagined and actually executed errors, where the overall profile of imagined and executed spatial errors do not closely overlap (Dahm and Rieger 2019; Roberts et al. 2020). This discrepancy alludes to a source of error that is perhaps less susceptible to conscious awareness, and more seamlessly unfolds following covert motor processes (van Beers 2009). With this in mind, it has been suggested that imagery may alternatively involve an abstract/amodal construct that operates outside of the motor system (Pylyshyn 2002). In a none-too-dissimilar vein, the more recent motor-cognitive model proposes that while imagery uses the same neural representation as execution for the initial programming of a movement, it is only later within the movement itself that an image has to be consciously formed due to the absence of actual sensory feedback (Glover and Baran 2017; Glover et al. 2020).

At this juncture, we may question whether there is an alternative and perhaps more definitive way to examine the potential representation of spatial characteristics within motor imagery. Specifically, while there is an absence of a physical movement that is required to directly capture the spatial trajectory within real time, it may be possible to capture the unintended or inadvertent influence of an imagined spatial trajectory (trial *n*-1) on a subsequent executed movement (trial *n*). This logic is adapted from the classic trajectory priming paradigm, where the higher trajectory following the presence of an obstacle compared to no obstacle within trial *n*-1 can cause the subsequent trajectory to shift higher within trial *n* (Jax and Rosenbaum 2007, 2009) (see also, van der Wel et al. 2007). This finding may be explained by the remnants or persistence of a motor program from one movement attempt at trial *n*-1 spilling over onto another attempt at trial *n*. Along these lines, the mere observation of a model spatial trajectory with or without an obstacle within trial *n*-1 can cause a corresponding shift of the trajectory of an executed movement within trial *n* (Griffiths and Tipper 2009, 2012). In this instance, it is suggested that the model observation activates a motor program at trial *n*-1 in much the same way as in execution, which then causes it to spill over at trial *n*.

To this end, the aim of the present study was to examine the potential representation of the spatial trajectory within motor imagery. In order to achieve this, we adapted the trajectory priming paradigm where imagery takes place at trial *n*-1 followed by execution at trial *n*. Of interest, there has been at least one study involving priming with imagery, which involved grasping objects of varying affordances (Glover and Dixon 2013). However, the primed characteristic in this instance pertained to grip selection, and was not necessarily related to the spatial trajectory that is of most interest to the present study. Hence, in the present study, we had participants either execute or imagine an aiming movement toward a target, where there was an obstacle or no obstacle in the way (trial *n*-1). Participants would then execute the same aiming movement with either an obstacle or no obstacle (trial *n*). Therefore, trials were paired together according to whether there was an obstacle or no obstacle (i.e., N-N, O-O, N-O, O-N). In line with the standard trajectory priming effect (Jax and Rosenbaum 2007), it was hypothesized that the heightened movement over an obstacle compared to no obstacle following execution within trial *n*-1 would contaminate or cause a corresponding shift of the trajectory within trial *n* (as indicated by early angular deviation and maximum height). However, more importantly, it was hypothesized that if the representation underpinning motor imagery comprises the spatial trajectory, then we would encounter a similar pattern of trajectory priming, where the spatial characteristics of imagery within trial *n*-1 would evoke a corresponding shift of the trajectory of an executed movement within trial *n*.

## Method

### Participants

An initial power analysis was conducted using G*Power software (version 3.1.9.4) (Faul et al. 2007) including the input parameters of *α* = 0.05, 1-*β* = 0.90, and *f* = 0.40 (large). The estimated effect size was directly adapted from previous studies surrounding trajectory priming (based on a main effect of trial *n*-1 for early angular deviation [Jax and Rosenbaum 2007] and maximum height [Griffiths and Tipper 2009, 2012]). Consequently, there was an estimated minimum number of 13 participants. For the actual study, there were 16 participants that initially volunteered, although 3 participants were removed due to recording error or issues in the motion capture (*n* = 13; 7 females and 6 males, self-reported right-handed, age range = 19–24 years, no known previous experience with the task, nor directed imagery).

Prior to commencing the study, participants provided written informed consent and completed the Movement Imagery Questionnaire-Revised (MIQ-R) (Hall and Martin 1997). This questionnaire involves an 8-item inventory on the visual (*M* = 22 / 28, *SE* = 1.39) and kinaesthetic (*M* = 17 / 28, *SE* = 1.30) imagery ability surrounding elementary bodily actions including knee lift, jump, horizontal shoulder abduction/adduction, and toe touch. The study was designed and conducted in accordance with the 1964 Declaration of Helsinki, and approved by the local research ethics committee.

### Task and materials

The main task involved a two-dimensional discrete aiming movement using a desk-mounted graphical digitizing tablet with a wired stylus pen (GTCO Calcomp Drawing Board VI; temporal resolution = 125 Hz, spatial resolution = 1000 lines per inch), which was occluded from view courtesy of an adjustable shelving unit. The subsequent position-time series data were transmitted and stored on an adjacent computer courtesy of a serial port connection that was controlled using Matlab (version 2018b) (The Mathworks Inc., Natick, MA). These data were used in real-time to virtually translate the limb position to a 17″ CRT monitor (spatial resolution = 1280 × 1024 pix, temporal resolution = 85 Hz) that was placed directly in front of participants (approx. 50-cm viewing distance). The display featured a left-sided circle (15-mm width) that represented the start, free-moving cursor dot (8-mm width) that represented the limb, and right-sided circle (15-mm width) that represented the target. The separation between the start and target objects assumed a 120-mm movement amplitude (centre-to-centre). On occasion, there was also a rectangular obstacle (30-mm width, 45-mm height) in the centre of the space between the start and target objects, which participants had to avoid by moving over it (for details, see *Procedures*). The stimuli were generated through Matlab running Psychtoolbox (version 3.0.11).

The aiming movement of interest was either physically executed or mentally imagined. The related procedures were consistent with the guidelines for reporting action simulation studies (GRASS) (Moreno-Verdú et al. 2024) (see Appendix 1). That is, participants were instructed using a standard script to physically move or imagine themselves see (1st person visual imagery) and feel (kinaesthetic imagery) a movement from the start position toward the target as quickly and accurately as possible (i.e., ‘motor imagery’; see Moreno-Verdú et al. 2024). For execution, the start and end of the movement was signalled by participants physically moving the stylus pen (represented by a cursor dot) from the first moment out of the start position and into the target while staying there for at least 50 ms (5 frames based on the screen refresh rate), respectively. For imagery, the start and end of the movement was signalled by participants pressing down and releasing the stylus tip, respectively, while remaining physically over the start position. In this regard, the imagery was made to closely simulate the execution task condition, but without any overt physical movement (e.g., PETTLEP model; see Holmes and Collins 2001; Wakefield et al. 2013).

### Procedure

**Fig. 1:**
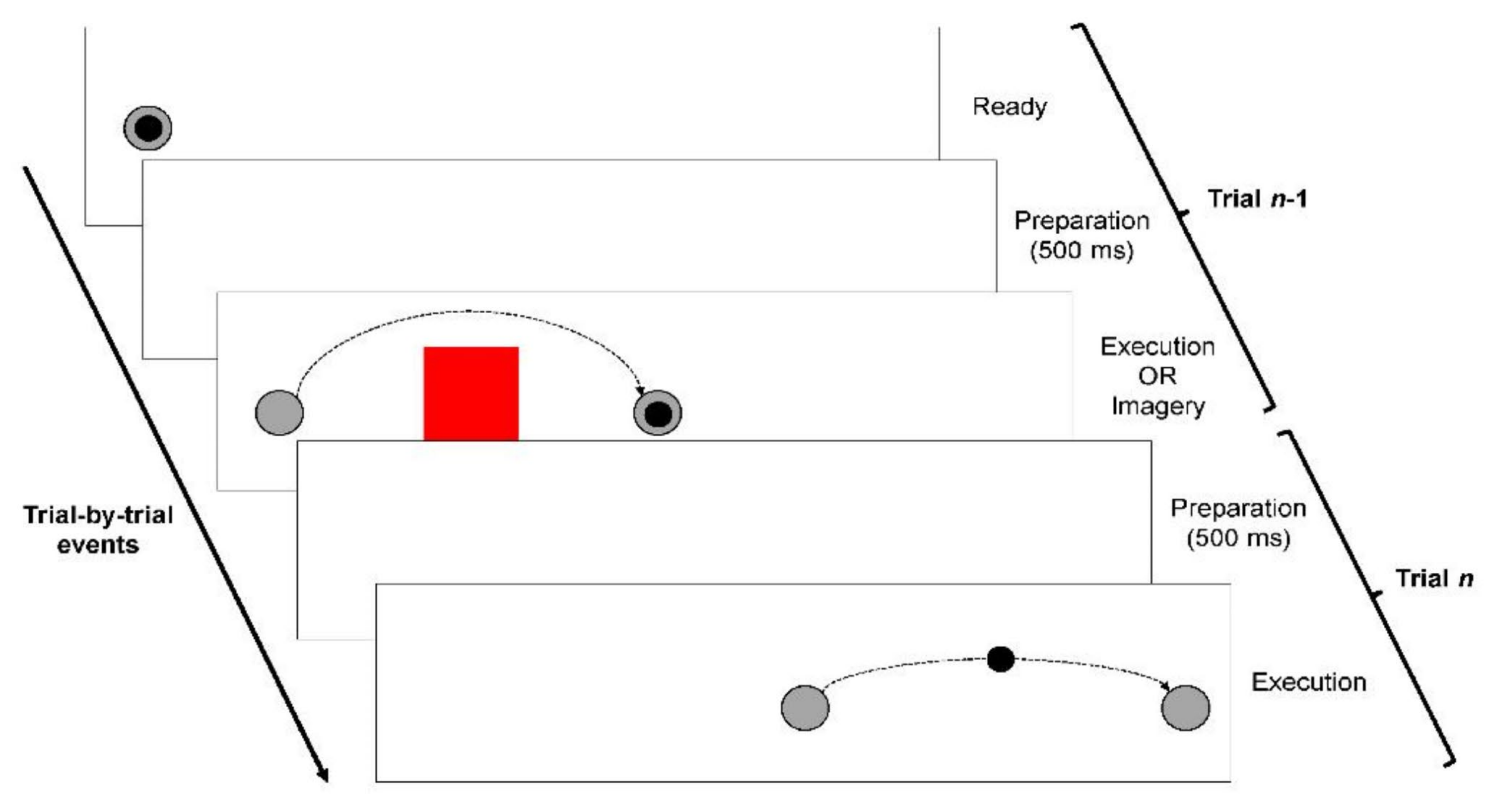
Trial-by-trial procedure. Following a 500-ms interval over the start position, the target appeared with or without an obstacle to trigger the participant to physically execute or mentally imagine themselves aiming (trial *n*-1). Upon movement termination, the previous objects disappeared and following another 500-ms interval, then a new target with or without an obstacle appeared to trigger participants to physically execute aiming (trial *n*). The present example illustrates an O-N trial pair involving execution in both trial n-1 and trial n

Individual consecutive trials were paired together (trial *n*-1 and trial *n*), where execution or imagery would unfold in trial *n*-1 and execution alone would unfold in trial *n* (Fig. 1). Specifically, if trial *n*-1 involved execution and the limb landed over the target position, then the next target for trial n would appear in a new position further over to the right. However, if trial *n*-1 involved imagery and the limb remained located over the start position, then the next target for trial *n* would appear in the same position as before on the right. The start position from trial *n*-1 would appear once more following the completion of trial *n* in order to start a new trial pairing.

Paired trials featured a unique combination of no obstacle and obstacle. In this regard, they were presented in either a constant (e.g., N-N, O-O) or alternate (e.g., N-O, O-N) fashion (Griffiths and Tipper 2009). In order to reasonably facilitate trajectory priming as a result of the obstacle manipulation, then the inter-trial interval between paired trials was selected at 500 ms (Jax and Rosenbaum 2009).

The execution and imagery protocols were counter-balanced across participants, while the different combinations of no obstacle and obstacle that formed the paired trials were randomized without replacement so that each possible combination appeared in every 4 paired trials. There was a familiarisation or practice at the beginning of each of the execution and imagery protocols, including 2 repetitions of each of the paired trial combinations (8 paired trials). For the experiment for real, the execution and imagery protocols went on to feature a further 5 repetitions of each of the paired trial combinations (20 paired trials) (for trial number recommendations, see Blinch et al. 2018; and De Grosbois et al. 2018).

### Dependent measures and analysis

To evaluate whether imagery was actively undertaken in trial *n*-1, we calculated reaction time and movement time using the originally collected data that marked the start and end of a trial (unfiltered). Specifically, reaction time was taken as the time difference between trial onset, and first moving out of the start position (execution) or pressing down the stylus tip (imagery). Meanwhile, movement time was taken as the time difference between the very first moments of moving out of the start position and into the target while staying there for at least 50 ms (5 frames) (execution), or pressing down the stylus tip and releasing it once again (imagery).

For the evaluation of trajectory priming, a more detailed kinematic analysis was warranted. Therefore, the raw position-time series data from trial *n* (execution) were smoothed using a 2nd -order, dual-pass Butterworth filter with a 10 Hz cut-off frequency. Therein, we adapted previous measures of trajectory priming including early angular deviation and maximum height (Jax and Rosenbaum 2007). The early angular deviation was taken as the signed angle of movement direction (i.e., positive values synonymous with an upward direction) with respect to the start position at 150 ms following movement onset (19 samples based on the digitizer sampling rate). This time was selected because it can be typically attributed to pre-response planning and occupies the more ballistic portion of the movement (Elliott et al. 2001, 2017). Meanwhile, the maximum height was simply taken as the peak orthogonal position from the entire movement trajectory.

Trial pairs (trial *n*-1 and trial *n*) were removed when at least one of the trials in question involved either a false start (i.e., < 100 ms reaction time), failed attempt to avoid an obstacle when present (i.e., < 20 mm maximum height) or an incomplete trajectory (4.81%). To evaluate imagery in trial *n*-1 independent of trial *n*, we first conducted a two-way fully repeated measures ANOVA, including factors of protocol (execution, imagery) and obstacle (no obstacle, obstacle) for reaction time and movement time data in trial *n*-1. To evaluate trajectory priming within trial *n*, we next conducted a three-way fully repeated-measures ANOVA, including factors of protocol, trial *n*-1 (no obstacle, obstacle) and trial *n* (no obstacle, obstacle) for movement time, early angular deviation and maximum height data in trial *n*. Significance was declared at *p* <.05, and the effect size measure of interest was partial eta-squared (*ƞ*_*p*_^*2*^). In the event a significant interaction, pairwise comparisons were subsequently drawn using the Holm-Bonferroni post hoc procedure.

## Results

### Imagery at trial n-1

Table 1 shows the mean reaction and movement time. For reaction time, there was a significant main effect of protocol, *F*(1,12) = 22.30, *p* <.001, *ƞ*_*p*_^*2*^ = 0.65, indicating a shorter time to initiate for execution (*marginal M* = 388 ms, *SE* = 21) compared to imagery (*marginal M* = 828 ms, *SE* = 105). There was no significant main effect of obstacle, *F*(1,12) = 0.22, *p* =.655, *ƞ*_*p*_^*2*^ = 0.02, nor a significant protocol x obstacle interaction, *F*(1,12) = 1.64, *p* =.225, *ƞ*_*p*_^*2*^ = 0.12.

For movement time, there was a significant main effect of protocol, *F*(1,12) = 18.57, *p =*.001, *ƞ*_*p*_^*2*^ = 0.61, indicating a shorter time within the movement for execution (*marginal M* = 733 ms, *SE* = 35) compared to imagery (*marginal M* = 1143 ms, *SE* = 95). In addition, there was a significant main effect of obstacle, *F*(1,12) = 44.28, *p* <.001, *ƞ*_*p*_^*2*^ = 0.79, indicating a shorter time for no obstacle (*marginal M* = 843 ms, *SE* = 50) compared to obstacle (*marginal M* = 1033 ms, *SE* = 60). However, there was no significant protocol x obstacle interaction, *F*(1,12) = 0.01, *p* =.925, *ƞ*_*p*_^*2*^ = 0.00.

**Table 1:** Mean (± SE) reaction and movement time (ms) for execution and imagery within trial *n*-1

Protocol	Execution		Imagery		
Obstacle	O	*N*	O	*N*	
Reaction Time	377 (21)	398 (23)	833 (104)	822 (107)	
Movement Time	826 (46)	639 (37)	1239 (107)	1046 (85)	

### Spatial trajectory priming at trial n

For movement time, there was a significant main effect of trial *n*, *F*(1,12) = 43.75, *p* <.001, *ƞ*_*p*_^*2*^ = 0.79, indicating a shorter time for no obstacle (*marginal M* = 656 ms, *SE* = 47) compared to obstacle (*marginal M* = 859 ms, *SE* = 61) (Table 2). Meanwhile, there was a significant trial *n*-1 x trial *n* interaction, *F*(1,12) = 11.16, *p =*.006, *ƞ*_*p*_^*2*^ = 0.48, although the shorter time in trial *n* with an obstacle following trial *n*-1 with no obstacle (*marginal M* = 830 ms, *SE* = 57) compared to obstacle (*marginal M* = 888 ms, *SE* = 66) failed to reach significance (*p* =.032, Holm-Bonferroni corrected alpha level = 0.025). There were no other significant main, nor interaction effects involving the factor of protocol [protocol: *F*(1,12) = 0.22, *p =*.648, *ƞ*_*p*_^*2*^ = 0.02; protocol x trial *n*-1: *F*(1,12) = 1.95, *p =*.188, *ƞ*_*p*_^*2*^ = 0.14; protocol x trial *n*: *F*(1,12) = 4.11, *p =*.065, *ƞ*_*p*_^*2*^ = 0.26; protocol x trial *n*-1 x trial *n*: *F*(1,12) = 0.60, *p =*.453, *ƞ*_*p*_^*2*^ = 0.05].

**Table 2:** Mean (± SE) movement time (ms) within trial *n* following execution and imagery at trial *n*-1 with an obstacle (O) or no obstacle (N)

Protocol	Execution				Imagery				
Trial *n*-1	O		*N*		O		*N*		
Trial *n*	O	*N*	O	*N*	O	*N*	O	*N*	
Movement Time	851 (59)	634 (46)	800 (46)	714 (57)	925 (80)	628 (53)	859 (75)	648 (55)	

**Fig. 2:**
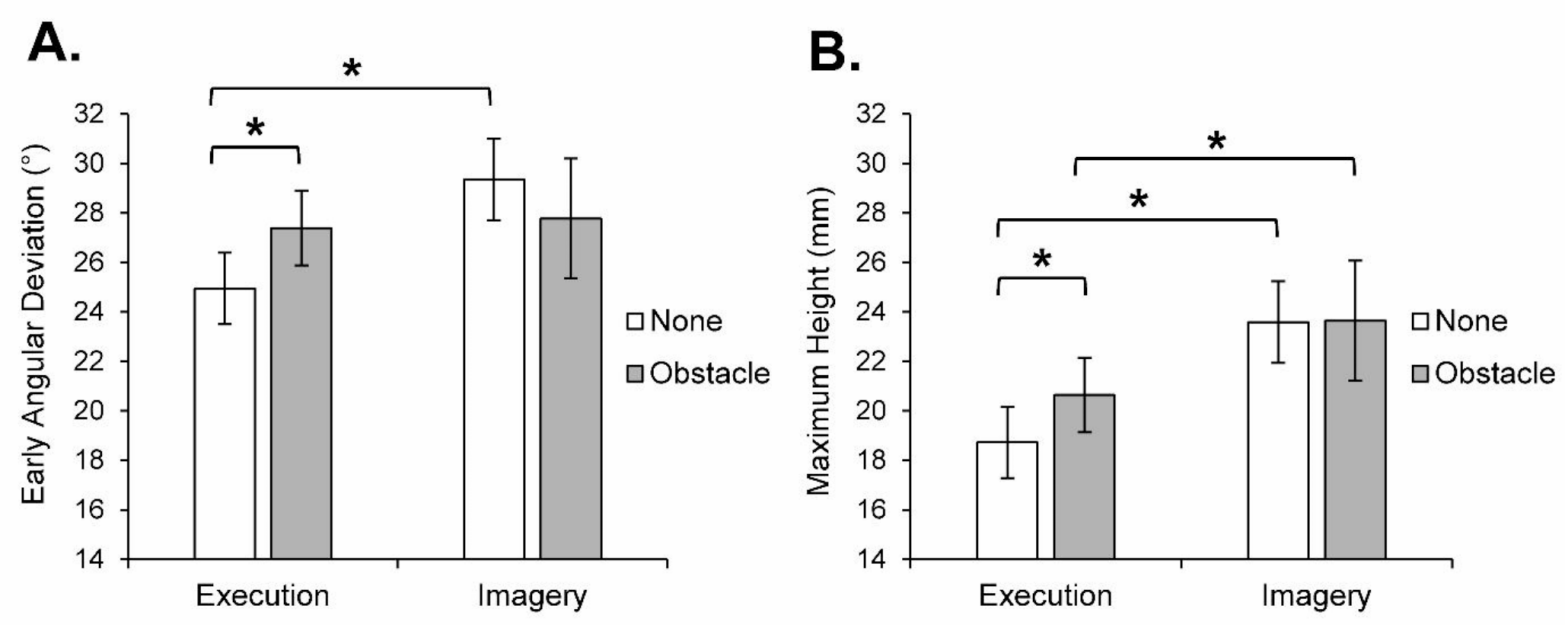
Mean early angular deviation (**A**) and maximum height (**B**) as a function of protocol and trial *n*-1 (see legend). Error bars indicate the standard error of the mean. (*) indicates a significant difference in a pairwise comparison (Holm-Bonferroni corrected)

For early angular deviation, there was a significant main effect of trial *n*, *F*(1,12) = 356.95, *p <*.001, *ƞ*_*p*_^*2*^ = 0.97, indicating significantly less deviation for no obstacle (*marginal M* = 2.52°, *SE* = 4.22) compared to obstacle (*marginal M* = 52.22°, *SE* = 2.81). Meanwhile, there was a significant protocol x trial *n*-1 interaction, *F*(1,12) = 5.81, *p* =.033, *ƞ*_*p*_^*2*^ = 0.33 (Fig. 2a), indicating significantly less deviation following execution at trial *n*-1 with no obstacle compared to obstacle (*p* =.0166, Holm-Bonferroni corrected alpha level = 0.017), while there was no such difference following imagery at trial *n*-1 (*p* =.26). In addition, there was significantly less deviation following execution compared to imagery at trial *n*-1 with no obstacle (*p* =.001, Holm-Bonferroni corrected alpha level = 0.013). There were no other significant main, nor interaction effects involving the factor of protocol [protocol: *F*(1,12) = 3.37, *p* =.091, *ƞ*_*p*_^*2*^ = 0.22; protocol x trial *n*: *F*(1,12) = 0.05, *p* =.823, *ƞ*_*p*_^*2*^ = 0.00; protocol x trial *n*-1 x trial *n*: *F*(1,12) = 3.61, *p* =.082, *ƞ*_*p*_^*2*^ = 0.23].

For maximum height, there was a significant main effect of protocol, *F*(1,12) = 24.52, *p <*.001, *ƞ*_*p*_^*2*^ = 0.67, and trial *n*, *F*(1,12) = 356.95, *p <*.001, *ƞ*_*p*_^*2*^ = 0.97. Meanwhile, there was a significant protocol x trial *n*-1 interaction, *F*(1,12) = 6.87, *p* =.022, *ƞ*_*p*_^*2*^ = 0.36 (Fig. 2b), indicating a significantly shorter height following execution at trial *n*-1 with no obstacle compared to obstacle (*p* =.004, Holm-Bonferroni corrected alpha level = 0.017), while there was no such difference following imagery at trial *n*-1 (*p* =.094). In addition, there was a significantly shorter height following execution compared to imagery at trial *n*-1 with no obstacle (*p* <.001, Holm-Bonferroni corrected alpha level = 0.013) and obstacle (*p* =.009, Holm-Bonferroni corrected alpha level = 0.025). Finally, there was a significant protocol x trial *n* interaction, *F*(1,12) = 9.72, *p* =.009, *ƞ*_*p*_^*2*^ = 0.45, indicating a significantly shorter height following both execution (*p* <.001, Holm-Bonferroni corrected alpha level = 0.013) and imagery (*p* <.001, Holm-Bonferroni corrected alpha level = 0.017) at trial n with no obstacle (execution: *marginal M* = -2.46 mm, *SE* = 0.88; imagery: *marginal M* = 3.54 mm, *SE* = 0.67) compared to obstacle (execution: *marginal M* = 41.84 mm, *SE* = 2.06; imagery: *marginal M* = 43.69, *SE* = 2.06), while there was significantly shorter height following execution compared to imagery at trial *n* with no obstacle (*p* <.001, Holm-Bonferroni corrected alpha level = 0.025). There was no significant protocol x trial *n*-1 x trial *n* interaction, *F*(1,12) = 2.31, *p* =.154, *ƞ*_*p*_^*2*^ = 0.16.

In the absence of trajectory priming following imagery at trial *n*-1, it is possible that some participants had poor imagery ability. If so, then we would anticipate a relation between trajectory priming and visual and/or kinaesthetic imagery ability. Therefore, we conducted a supplementary analysis by correlating the difference between obstacle and no obstacle at trial *n*-1 with imagery ability scores. For early angular deviation, there was no significant relation for kinaesthetic, *r*(13) = − 0.06, *p* =.86, nor visual, *r*(13) = − 0.10, *p* =.75, imagery ability. For maximum height, there was no significant relation for kinaesthetic, *r*(13) = 0.46, *p* =.11, but there was for visual, *r*(13) = 0.64, *p* =.02, imagery ability. Further inspection of the latter correlation indicated that it was primarily attributed to a single case, which once removed indicated that there was no longer a significant relation, *r*(12) = 0.39, *p* =.21.

## Discussion

The present study aimed to examine whether the spatial trajectories are represented within motor imagery. Participants were tasked with the execution or imagery of aiming to a target (trial *n*) followed by execution to another target (trial *n*-1) with an obstacle or no obstacle in the way. In line with the standard trajectory priming effect (Jax and Rosenbaum 2007), it was expected that the higher trajectory needed to actively avoid an obstacle when in execution at trial *n*-1 would cause a similarly higher shift in the trajectory at trial *n*. If the spatial trajectory comprises the representation for motor imagery, then we anticipated a similar pattern of trajectory priming following imagery at trial *n*-1.

The findings indicated a higher trajectory following execution at trial *n*-1 with an obstacle compared to no obstacle (Jax and Rosenbaum 2007, 2009) (see also, van der Wel et al. 2007). While there was a trajectory priming effect in execution, there was no such difference following imagery at trial *n*-1 between the obstacle and no obstacle. Therefore, it appears the spatial trajectory may hold a limited representation within motor imagery. Despite arguably conflicting with the concept of equivalence (Jeannerod 1994, 1999), where motor imagery and execution supposedly use the same or a common neural representation, it may be that the equivalence is limited to particular features of the movement. For example, there are a plethora of findings featuring the mental chronometry paradigm, where there is a relatively close match between execution and imagery when it comes to the relation between task difficulty and movement time (Guillot et al. 2012). Instead, it may be that imagery fails to adequately capture the spatial trajectory because there is an absence of sensory feedback from the movement that would otherwise take place during execution. The absence of this sensory feedback may prohibit imagery to such an extent that additional processes are needed for it to be upheld or continue. For example, it is suggested that imagery may be at least partially compensated by accessing executive resources to help consciously form and monitor an image (Glover and Baran 2017; Glover et al. 2020) (see also later within *Discussion*). Alternatively, imagery may be simultaneously combined with the observation of a model (Wright et al. 2018; Bruton et al. 2020; Romano-Smith et al. 2019, 2022; see also, Campos et al. 2009), where the subsequent visual afference can help “guide” the image (Meers et al. 2020).

Upon review, it could be argued that the absence of a trajectory priming effect in imagery may simply be the result of a failure by participants to positively or vividly engage in motor imagery. Indeed, this possibility is an often lingering one when it comes to finding a discrepancy or failing following motor imagery (e.g., Roberts et al. 2020), where there is no direct means to precisely capture whether imagery even unfolded, and thus the participant is primarily held responsible for the experimental control. However, our findings indicated a longer reaction and movement time for imagery compared to execution within trial *n*-1. The finding for reaction time aligns with previous other studies that suggest motor imagery involves a greater emphasis on pre-response planning (Owen et al. 2024), and/or an anticipated absence of any upcoming sensory feedback (Khan et al. 2002; Hansen et al. 2006; Roberts and Bennett 2022). Likewise, the finding for movement time is consistent with widespread evidence of a more prolonged time to terminate the movement for imagery (Guillot et al. 2012) owing to it accessing an under-developed or un-adapted motor programme (Yoxon et al. 2015), and/or the need for executive resources to consciously form and monitor an image (Glover and Baran 2017; Glover et al. 2020). Therefore, in this instance, we can be confident that motor imagery initially took place within trial *n*-1, while the apparent failure to reveal a trajectory priming effect following imagery simply alludes to it not comprising of the spatial trajectory.

Perhaps surprisingly, there was an overall higher trajectory following imagery compared to execution at trial *n*-1 independent of any prime context (i.e., obstacle). While this heightened trajectory may have indicated a ceiling effect that limited the capacity to capture any priming related to the obstacle (i.e., obstacle > no obstacle), it may also allude to a failure in updating the motor programme across a series of trials. That is, participants may have adopted a conservatively high trajectory throughout the imagery protocol with a view to there being the possibility of an obstacle. In this regard, they may have traded off the biomechanical cost of a higher trajectory in favour of avoiding a more taxing and perhaps compromising switch between movement strategies in imagery (trial *n*-1) followed by execution (trial *n*) (Poletti et al. 2015, 2016). This suggestion may resonate once again with the motor-cognitive model (Glover and Baran 2017; Glover et al. 2020), where if executive resources are to be more greatly used for consciously forming and monitoring an image later on within the movement, then it stands to reason that they repeatedly adopt a strategy that limits any cost to such resources.

However, at this juncture, it is important to consider the potential limitations of the present study. Indeed, the sample size was comparatively limited, the slightly different start locations between the execution and imagery protocols may have confounded trial *n* effects, and the higher overall trajectories following imagery may have undermined any potential influence of the obstacle. With this in mind, and because at least some of these limitations were unavoidable, it may be useful for future research to alternatively capture the spatial characteristics of motor imagery within trials, as opposed to between trials like the present study. For example, our lab is currently undertaking an interference paradigm, where individuals simultaneously imagine and execute movements that have an incongruent spatial trajectory (see also, Piedmonte et al. 2018). To elucidate, if imagery does sufficiently represent the spatial characteristics of movement, then we would anticipate some interference or unintended deviation in the executed movements.

In summary, the present study offers one of few empirical accounts to more directly capture the spatial characteristics of motor imagery. That is, we highlight how the trajectory priming effect that is mediated by the presence or absence of an obstacle across consecutive trials, fails to unfold following imagery as it does execution. As it stands, we take it that motor imagery holds a limited representation of the spatial trajectory owing to an absence of sensory feedback from executed movement.

## Electronic supplementary material

Below is the link to the electronic supplementary material.

Supplementary Material 1

## Data Availability

Data will be made available from the first/corresponding author upon request.
