# Supplementary material for: Trajectory priming through obstacle avoidance in motor imagery – does motor imagery comprise the spatial characteristics of movement?
Source: Exp Brain Res. 2024 Dec 2;243(1):9. doi: 10.1007/s00221-024-06951-3 (PMC11609121; doi:10.1007/s00221-024-06951-3)
Supplement: Supplementary file 1 — Supplementary Material 1 [file 221_2024_6951_MOESM1_ESM.docx]

**G**uidelines for **R**eporting **A**ction **S**imulation **S**tudies (GRASS)

Checklist

*Page references taken with respect to the original pre-print version

**Part A: Essential items for general study reporting**

| # | Item | Pages |
| --- | --- | --- |
| A1 | Are participant characteristics (age, sex, handedness, experience with similar tasks, vision, clinical details, etc) included for the final study sample/groups? | 5-6 |
| A2 | What instructions were provided? How were they delivered (spoken, written, etc)? | 7 |
| A3 | Were standard instructions used (i.e. a script, information sheet etc)? Is this available to readers (in the manuscript, supplementary materials, an online repository, etc)? | 7 |
| A4 | Was adherence to instructions monitored (e.g. EMG recordings, post test questionnaires, repeated instructions, manipulation checks, etc)? | 7 |
| A5 | Do statistical comparisons include the average and standard deviation or standard error of the mean for the groups/conditions? | 10-13 |
| A6 | Is the ‘dose’ used in the study clearly defined (i.e. sessions, blocks, trials, duration, etc)? | 8 |

**Part B: Essential items relating specifically to motor imagery**

| # | Item | Pages |
| --- | --- | --- |
| B1 | Were participants instructed to use kinesthetic imagery, visual imagery, or a combination of both? | 7 |
| B2 | If visual imagery was used, is the visual perspective (1st person, 3rd person) stated? | 7 |
| B3 | If 3rd person imagery was used, is the vantage point specified? Is it illustrated? | N/A |
| B4 | Were participants previously familiar with motor imagery (e.g. sports practice, prior participation in experiments)? | 6 |

**Part C: Discretionary Items (to be included as appropriate on a case-by-case basis)**

| # | Item | Pages |
| --- | --- | --- |
| C1 | Are study materials/data/code openly available (including a link to a repository)? | N/A |
| C2 | Were imagery instructions based on a framework (e.g. PETTLEP, LSRT)? If so, how? | 7 |
| C3 | Was imagery ability/quality assessed (e.g. questionnaires, chronometry)? | 6, 10-11 |
| C4 | Was the participant’s body posture matched with the action(s) they imagined (e.g. were imagined/actual postures matched, mirrored, etc)? | 7 |
| C5 | Was movement during imagery instructed/allowed (e.g. dynamic motor imagery)? | 7 |
| C6 | Were other modalities of imagery (e.g. auditory, haptic, olfactory, gustatory) instructed or reported by participants? | 7 |
